# Barriers and facilitators for interaction in cardiopulmonary resuscitation teams: a qualitative study

**DOI:** 10.1186/s12245-025-00955-8

**Published:** 2025-07-28

**Authors:** Abdolhosein Emami Sigaroudi, Nazila Javadi-Pashaki, Mohammad Ali Cheraghi, Yadolah Shirvani

**Affiliations:** 1 Department of Community Health Nursing, School of Nursing and Midwifery, Guilan University of Medical Sciences, Rasht, Iran; 2 Social Determinants of Health Research Center, Guilan University of Medical Sciences, Rasht, Iran; 3 Department of Nursing Management, School of Nursing and Midwifery, Tehran University of Medical Sciences, Tehran, Iran; 4 Department of Nursing, School of Nursing and Midwifery, Guilan University of Medical Sciences, Rasht, Iran; 5 Department of Cardiology, Cardiovascular Diseases Research Center, Heshmat Hospital, School of Medicine, Guilan University of Medical Sciences, Rasht, Iran

**Keywords:** Cardiopulmonary resuscitation, Teamwork, Cardiac arrest, Qualitative study

## Abstract

**Introduction:**

Cardiac arrest is a life-threatening emergency whose outcome depends on immediate interventions, known as cardiopulmonary resuscitation (CPR). The quality of these interventions hinges on the performance and communication of the resuscitation team. Therefore, this study aimed to explore factors affecting interactions among CPR team members.

**Methods:**

This qualitative study employed a content analysis approach conducted in Iran over a 12-month period from December 2023 to December 2024. The study population included all CPR team members at academic medical centers. Researchers used purposive sampling and continued recruitment until data saturation. Data collection involved conducting in-depth, semi-structured interviews; all data were analyzed using MAXQDA software (version 20).

**Results:**

Data analysis revealed one main category entitled “The Complexity of Cardiopulmonary Resuscitation Interactions,” along with 5 general categories and 11 subcategories: “Consensus in Resuscitation” (including “Pre-resuscitation Coordination” and “Post-resuscitation Debriefing”), “Communication Clarity” (comprising “Regular Communication” and “Irregular Communication”), “Interaction in Team Rotation” (with “Normal Rotation” and “Abnormal Rotation”), “Personal Conflicts” (featuring “Pre-Resuscitation Conflicts” and “Intra-Resuscitation Conflicts”), and “Team Leadership Style” (encompassing “Autocratic Leadership,” “Laissez-faire Leadership,” and “Participatory Leadership”).

**Conclusion:**

The results demonstrated that CPR team interactions were influenced by multiple factors. Through careful planning to enhance facilitating factors - including consensus in resuscitation, regular communication, normal rotation, and participatory leadership - while addressing inhibiting factors - such as irregular communication, personal conflicts, abnormal rotation, and autocratic leadership - we can optimize team interactions to improve CPR outcomes.

**Supplementary Information:**

The online version contains supplementary material available at 10.1186/s12245-025-00955-8.

## Introduction

Sudden cardiac arrest is one of the leading causes of death worldwide. In the United States, approximately 350,000 to 450,000 people experience cardiac arrest annually, while Europe reports about 700,000 cases each year [1, 2]. In Iran, cardiovascular diseases are responsible for 80% of mortality cases [3]. Patient outcomes following cardiac arrest depend strongly on the immediate and proper implementation of both basic and advanced cardiac life support measures, particularly cardiopulmonary resuscitation (CPR) [4]. Despite continuous updates to CPR guidelines, survival rates remain alarmingly low, ranging from just 6–9% [5]. Iranian studies indicate even more concerning statistics, with CPR mortality rates exceeding 90% and hospital discharge rates falling below 7% [3]. While recent medical advancements have modestly improved survival outcomes, these rates continue to be unsatisfactory [6–8].

CPR is one of the most stressful and chaotic procedures in healthcare settings [9]. Improving CPR quality is crucial, as it directly correlates with patient survival outcomes [10, 11]. Successful resuscitation requires coordinated team efforts, frequently involving professionals with diverse skill sets who may lack prior collaborative experience [4]. A healthcare team is formally defined as two or more individuals interacting dynamically, sharing common objectives, and contributing complementary competencies [12]. In high-acuity situations like CPR, technical proficiency alone proves inadequate; effective teamwork and non-technical skills become equally vital. Individual characteristics of resuscitation team members such as technical skills, previous experience, communication, and leadership skills influence the course of action during a resuscitation [13].

Teamwork is a process that facilitates interactions among team members through effective communication, collaboration, and coordination. It helps accomplish tasks successfully while fostering high-quality relationships [14]. Training and practicing teamwork skills improve the performance of nurses and physicians of the resuscitation team. Teamwork in various settings can be different, so studying teamwork in different settings and cultures is important [15]. Poor teamwork is responsible for almost 40% of medical malpractice cases [16].

According to the American Heart Association (AHA) guidelines, a resuscitation team typically consists of a team leader (physician in charge), an airway management provider, one or more chest compressors, a medication/IV access provider, a monitor/defibrillator operator, and a recorder/timekeeper [2]. While Iran follows this same team composition structure according to the AHA guidelines, In Iranian hospitals, the recorder/timekeeper role is typically omitted due to staffing shortages. Cardiopulmonary resuscitation (CPR) is one of the most critical medical procedures requiring coordinated teamwork among healthcare professionals. In this life-saving intervention, each team member has distinct responsibilities: physicians lead and direct the resuscitation while developing treatment plans; nurses perform cardiac compressions and administer medications, including defibrillation shocks; and anesthesia technicians establish airway access and provide mechanical ventilation [17].

According to a study by Hosseini et al., the category of team interaction elements included shared mental models, communication, cooperation, coordination, prioritization, and cognitive aids, with communication being the most frequent among these elements [18]. Studies indicate that 70–80% of healthcare errors stem from poor communication and team misunderstandings, underscoring the critical role of effective communication during the resuscitation of critically ill patients [19, 20]. The importance of communication in CPR has been extensively documented in the literature [21, 22]. Similarly, Rosenman et al. found that 50% of errors in trauma resuscitation were linked to teamwork and leadership failures [23].

In-hospital cardiac arrest (IHCA) represents a complex clinical scenario where CPR team members frequently have no prior collaborative experience [24]. In such high-stakes emergencies, successful outcomes rely not just on individual performance but on coordinated interactions among team members and systems [25]. To improve team performance during CPR, implementation of effective interaction strategies is essential. The objective of this study is to identify and explain the factors influencing interactions among CPR team members.

## Methods

### Study design and setting

This study employed a qualitative approach based on conventional content analysis. The study was conducted over 12 months (December 2023-December 2024) in government hospitals affiliated with Guilan University of Medical Sciences, including three general hospitals and one specialized heart center. To ensure maximum variation, we collected samples from different departments across all four centers, and used the Consolidated Criteria for Reporting Qualitative Research (COREQ) checklist to ensure quality. Content analysis was selected as the methodological approach for interpreting context-based data [26]. Specifically, we employed the Elo and Kyngäs method, which is particularly suitable for studying phenomena with limited available information [27]. To ensure trustworthiness, we applied Lincoln and Guba’s criteria [28]. The researcher made an attempt to increase the credibility of data by conducting in-depth interviews, making adequate interaction and cooperation with the participants, collecting credible data. We also performed member checking by asking participants to verify the collected data. To ensure dependability, we provided a detailed description of all study procedures, enabling readers to evaluate the research process accurately. To strengthen confirmability, we shared transcriptions, codes, and categories with other research team members for cross-validation of the coding process. To ensure transferability, we provided comprehensive details about the research process and participant characteristics, allowing others to assess the applicability of findings in different contexts.

### Participants

The study participants included all resuscitation team members: physicians, nurses, anesthesia experts, and residents. Using purposive sampling, we selected eligible participants from various departments to maximize diversity in clinical experience, gender distribution, and professional roles within CPR teams. The inclusion criteria required participants to: (1) be members of resuscitation teams at government hospitals affiliated with Guilan University of Medical Sciences, (2) have clinical experience in cardiac arrest management, (3) be actively involved in CPR operations, (4) demonstrate willingness to communicate effectively, (5) possess ability to articulate their experiences and perspectives, and (6) voluntarily participate in the study.

### Data collection

We used purposive sampling and continued data collection until reaching saturation. The concept of saturation is the most important factor in determining the sample size in qualitative studies. Data saturation refers to the time that the data collection process no longer provides any new or relevant data [29]. In our study, data saturation was achieved after conducting 15 semi-structured, face-to-face interviews. All interviews were conducted by a nursing PhD candidate with 20 years of practical experience in hospital resuscitation teams and specialized training in qualitative methods. In-depth interviews were conducted in quiet, private settings at times and locations chosen by participants to ensure comfort and confidentiality. Prior to each interview, written informed consent was obtained, and participants were assured of their right to pause or withdraw at any time. Following each interview, we analyzed the data before conducting the next session to obtain deeper insights into participants’ experiences through semi-structured interviews. We developed an interview guide based on key teamwork components identified in previous research (see Supplementary File), structuring the sessions around open-ended questions that allowed participants to share their perspectives in depth. Sample questions included: “What has been your experience with teamwork during cardiopulmonary resuscitation?“, “How would you describe interactions within your CPR team?“, and “What factors have you observed affecting CPR team interactions?” During the interviews, we incorporated exploratory follow-up questions to better understand participants’ beliefs and experiences, with all sessions being digitally recorded for accurate data collection.

### Data analysis

The data analysis followed the conventional qualitative content analysis method, which involves interpreting the content of the data based on personal perspectives. As soon as each interview finished we transcribed the audio recordings verbatim using Microsoft Word, then analyzed the transcripts using MAXQDA 20 software. The research team extracted categories directly from the text data and identified codes and themes through a systematic classification process. This method aims not only to extract objective content but also to uncover underlying themes and patterns within the participants’ responses. The analysis started with several reviews of the interview transcripts. We implemented a structured analytical sequence: (1) open coding, (2) indexing, (3) grouping, (4) categorizing, and (5) abstracting. Accordingly, through the analysis process, the analysis units, which can be analyzed and coded, were selected from the interviews, and were categorized into smaller units including the meaning units, the codes (which include the title of coding for the meaning units), the categories (which include a group of content with meaning and conceptual commonality), and the main category.

## Results

Over the 12 months, 15 interviews were conducted with 14 participants, as detailed in Table 1.

**Table 1:** Characteristics of the participants

Participant	Age	Gender	Occupation	Education	Experience Duration	First Interview Duration	Second Interview Duration
Cod 1	50	M	Nurse, Clinical Instructor	PHD	25	55	
Cod 2	52	F	ICU Nurse	Bachelor	26	47	
Cod 3	36	F	Cardiac Nurse	Bachelor	17	25	
Cod 4	53	F	Anesthesia expert	Bachelor	17	42	
Cod 5	25	M	Emergency Nurse	Bachelor	2	45	
Cod 6	38	F	Clinical Supervisor	Master	19	35	25
Cod 7	39	M	Cardiac Resident	Cardiac Resident	5	56	
Cod 8	38	M	Anesthesia expert	Bachelor	18	55	
Cod 9	45	M	Clinical Supervisor	Master	20	45	
Cod 10	55	M	General Physician	General Physician	20	30	
Cod 11	28	F	Emergency nurse	Bachelor	5	40	
Cod 12	53	M	physician	Emergency medicine specialist	23	42	
Cod 13	44	M	Clinical Supervisor	Master	24	33	
Cod 14	30	F	Surgical department nurse	Bachelor	13	40	

The research team achieved data saturation after completing 15 interviews, as subsequent analyses yielded no new codes. One participant was interviewed twice to clarify and expand on their initial responses. The interviews ranged from 25 to 56 min (M = 40), providing in-depth insights into participants’ experiences. Our study cohort included 14 healthcare professionals (6 women, 8 men) averaging 42 years of age (range 25–55), representing diverse clinical perspectives. Data analysis revealed a main category titled “The Complexity of Cardiopulmonary Resuscitation Interactions”. It also identified 5 general categories and 11 subcategories (Table 2). These categories and subcategories provided a structured understanding of the factors influencing CPR team interactions, highlighting both facilitators and barriers to effective teamwork during resuscitation efforts.

**Table 2:** The main, general and sub-categories of interaction in cardiopulmonary resuscitation teams

Main categories	General categories	Subcategories
The Complexity of Cardiopulmonary Resuscitation Interactions	Consensus in Resuscitation	Pre-Resuscitation Coordination
		Post-Resuscitation Debriefing
	Communication Clarity	Regular Communication Irregular Communication
	Interaction in Team Rotation	Normal Rotation
		Abnormal Rotation
	Personal Conflicts	Pre-Resuscitation Conflicts Intra-Resuscitation Conflicts
	Team Leadership Style	Autocratic Leadership
		Laissez-faire Leadership
		Participatory Leadership

### The complexity of cardiopulmonary resuscitation interactions

Team interactions during cardiopulmonary resuscitation (CPR) are influenced by various factors. While some factors enhance teamwork effectiveness, others pose challenges. These factors contribute to the complexity of team interactions. For participants, the barriers and facilitators of interaction held different meanings, significantly impacting their interaction, performance, and responses during resuscitation efforts.

## Consensus in resuscitation

“Consensus in Resuscitation” is a key dimension shaping the concept of the complexity of cardiopulmonary resuscitation interactions. Participants emphasized that consensus is critical for successful resuscitation. Team members should review and discuss teamwork deficiencies, how to perform effectively, and how to fulfill their roles both before and after CPR cases. These discussions should take place during training sessions or regular team meetings. Such reflective practices enable teams to improve their interactions during resuscitation and achieve well-coordinated, seamless role execution in CPR.

### Pre-Resuscitation coordination

The concept of “Pre-Resuscitation Coordination” is one of the dimensions of the concept of consensus in resuscitation, as participants emphasized the importance of holding prior coordination sessions to ensure a well-coordinated role during resuscitation. As noted by Participant No. 1, a nurse on the resuscitation team and a CPR instructor:

*“In the post-resuscitation team formation phase*,* we don’t have any meetings where we sit together to discuss what we should do if resuscitation occurs. That is*,* we don’t address the storming and norming stages that are part of the teamwork process.”*

Participant No. 3, a nurse with 20 years of experience in the cardiac department, highlighted the need for consensus in resuscitation sessions. She emphasized teaching team skills and coordination tips in training classes.

*“We didn’t have any consensus in resuscitation sessions before or after the resuscitation. They hold training classes for resuscitation*,* but they don’t teach the points that need to be considered for team coordination and the team’s duties; it’s just a theory class*,* and technical points are taught.”*

### Post-Resuscitation debriefing

“Post-Resuscitation Debriefing” represents one of the key dimensions within the concept of consensus in resuscitation. Participants emphasized the importance of these sessions to address skill gaps and improve performance in future resuscitations. As Participant No. 5, a cardiac emergency department nurse, explained:

*“We sometimes have friendly conversations about the outcome of resuscitation. However*,* we lack a structured*,* regular post-resuscitation meeting. Such a meeting could effectively address issues in teamwork and communication*,* helping us identify areas for improvement and ensuring better coordination in future cases.”*

## Communication clarity

“Communication Clarity” is another dimension that shapes the concept of the complexity of cardiopulmonary resuscitation interactions. Clear communication is crucial for effective resuscitation teamwork, as participants noted that it enables them to perform cardiopulmonary resuscitation tasks correctly and quickly. Team members must share information clearly and remain informed about the methods and processes involved in resuscitation.

### Regular communication

“Regular Communication” is one dimension of the concept of communication clarity. In the experiences of the participants, the importance of regular communication among the members of the cardiopulmonary resuscitation team and the timely, loud announcement of the actions taken was mentioned. Participant No. 2 (ICU nurse, 24 years’ experience), said in this regard:

*“A good leader regularly announces instructions loudly and clearly. Then*,* experienced members will also announce when they complete an action. I*,* who am experienced and understand the importance of this practice*,* do this consistently. However*,* unfortunately*,* this type of training is not provided to novices. For example*,* I will announce*,* ‘I am injecting the third dose of epinephrine*,*’ but our less experienced colleagues often fail to do this. This lack of communication can lead to improper information exchange*,* potentially resulting in the patient receiving additional or insufficient medication.”*

### Irregular communication

“Irregular Communication” is another dimension of the concept of communication clarity. Participants’ experiences indicated irregular communication and a lack of coordination during shock delivery, which impacted personnel safety. Participant No. 4, an anesthesia expert with 17 years of experience in the cardiac emergency department, said:

*“A few years ago*,* I experienced a situation where there was poor communication and coordination during shock delivery. The resident was supposed to deliver a shock and should have clearly announced it*,* but she didn’t. As a result*,* I was inadvertently exposed to the shock*,* which caused me to experience severe tachycardia. I had to leave the patient immediately*,* as I wasn’t feeling well at all.”*

## Interaction in team rotation

Concerning team rotation, “Interaction in Team Rotation” is a key component for understanding the complexity of cardiopulmonary resuscitation (CPR) interactions. Team members must interact to rotate roles, preventing ineffective performance and teamwork disruption. They must exchange information during role rotation and ensure critical tasks are handed over at optimal moments without disrupting resuscitation.

### Normal rotation

“Normal Rotation” is one of the dimensions of the concept of interaction in team rotation. Participants’ experiences highlight the importance of appropriate and timely rotation of resuscitation team members to prevent disorganization and avoid wasting valuable time during critical situations.

Participant No. 2 emphasized:

*“It is important to know who is responsible for which task during resuscitation. For example*,* if C takes over A’s or D’s role without proper coordination*,* team order will be disrupted. Typically*,* the medication and chest compression teams switch roles*,* but they must interact effectively to ensure that information about how many drugs and which ones have been administered is clearly communicated. This transfer of responsibilities must happen seamlessly and without wasting time to maintain the efficiency and effectiveness of the resuscitation effort.”*

### Abnormal rotation

“Abnormal Rotation” represents one dimension of the concept of interaction in team rotation and hinders effective team collaboration. Participants’ experiences highlight the critical need to avoid individualism in cardiac massage and to ensure the timely handover of roles among team members to sustain effective performance during resuscitation efforts. According to the American Heart Association (AHA) protocol, effective cardiac massage during cardiopulmonary resuscitation requires that team members performing chest compressions switch roles every 2 min, or before they become fatigued. This ensures that compressions remain consistent, high-quality, and effective throughout the resuscitation effort.

In this regard, Participant No. 1 highlighted a critical issue: the quality of resuscitation drops significantly if one person continues chest compressions for more than 2 min. They explained that this often stems from a feeling of negative competition during resuscitation. The participant noted:

*“When we ask someone to switch roles*,* they sometimes refuse*,* saying*,* ‘No*,* I’m not tired.’ But we know that continuing beyond 2 minutes leads to low-quality resuscitation. The individual may feel that stepping down early is a sign of weakness*,* so they resist. This creates a kind of negative competition*,* where the person thinks*,* ‘I’ll stay longer to prove I’m stronger"*.

## Personal conflicts

The fourth aspect of the complexity of cardiopulmonary resuscitation interactions is “Personal Conflicts”. The presence of personal conflicts among team members and the inability to work as a team in cardiopulmonary resuscitation can create significant barriers to establishing effective team interaction. These conflicts may hinder role performance during resuscitation and ultimately lead to the failure of teamwork in critical situations. Interpersonal conflicts may occur either before or during CPR team resuscitation efforts.

### Pre-Resuscitation conflicts

“Pre-Resuscitation Conflicts” represent one dimension of personal conflicts. Participants’ experiences highlighted the importance of avoiding pre-resuscitation conflicts to ensure orderly and coordinated teamwork among cardiopulmonary resuscitation (CPR) team members.

Participant No. 3 said in this regard:

*“Because the team members have not had prior meetings or consensus with each other*,* and sometimes they do not know each other*,* they may have unresolved conflicts from the past. These conflicts can prevent them from working together as a cohesive team during resuscitation.”*

Participant No. 9 emphasized the significance of prior conflicts and their effect on trust deficits among resuscitation team members, stating:

*“…For instance*,* there may be an argument between a nurse and the head nurse about shift scheduling the day before resuscitation. This can undermine mutual trust and cause delays in team coordination during resuscitation.”*

### Intra-Resuscitation conflicts

“Intra-Resuscitation Conflicts” is one of the dimensions of the concept of personal conflicts. The participants’ experiences emphasized the importance of prior acquaintance among team members, avoiding interpersonal conflicts, and maintaining effective task performance during resuscitation. Participant No. 1 said in this regard:

*“As members of the resuscitation team*,* we may not see each other until just before the resuscitation procedure. We often cannot determine whether we have the ability to work effectively as a team with these colleagues. For example*,* I might suddenly realize at 11 AM that I’m part of the CPR team*,* joined by one member from the women’s CCU and another from the men’s CCU. In such cases*,* we might experience conflicts that could prevent us from working together effectively.”*

Participant No. 14 emphasized the importance of friendly relations among cardiopulmonary resuscitation team members for coordinated teamwork, stating:

*“Since we had already worked together and established friendly relationships*,* our interactions during resuscitation were effective. We could comfortably say*,* “Doctor*,* you might not want to do this*,*” or “So-and-so*,* please do this.” However*,* personnel unfamiliar with each other tend to be more reserved*,* which may hinder their ability to function well as a team in such stressful situations.”*

## Team leadership style

The fifth feature of the complexity of cardiopulmonary resuscitation interactions is “Team Leadership Style.” Data analysis revealed that resuscitation teams employ different leadership styles to facilitate interaction and coordination during cardiopulmonary resuscitation (CPR). The dimensions of this concept include: autocratic leadership, laissez-faire leadership, and participatory leadership.

### Autocratic leadership

“Autocratic Leadership” is one of the dimensions of the concept of team leadership style. Participants’ experiences highlighted the leaders’ one-sided approach and their tendency to act based on personal preferences during cardiopulmonary resuscitation (CPR), which often leads to the suppression of team members’ participation in decision-making.

Participant No. 2 said in this regard:

*“Sometimes we say*,* ‘Doctor*,* the patient’s rhythm is shockable. Should we shock them?’ And the doctor agrees. Other times*,* they act based on their own preference and say*,* ‘No*,* give the medication for now*,*‘…"*.

Participant No. 1 also pointed out the individualism of the team leader, who often tries to prove their versatility and said:

*“In CPR*,* there is a tendency for individualism*,* especially among team leaders. Some individuals may want to create the impression that they are all-capable*,* that they can handle everything. They don’t give other team members the opportunity to contribute or share their input*,* which leads to others realizing over time that they have little role in decision-making. As a result*,* team members gradually become suppressed.”*

### Laissez-faire leadership

“Laissez-faire Leadership” represents another dimension within the concept of team leadership styles. Field notes highlight the lack of supervision over team performance and coordination, as well as the leader’s indifference toward the actions taken during cardiopulmonary resuscitation (CPR).

Field Note No. 1 provides an example:

*“The team leader’s communication with the members was weak. They were indifferent to reminding team members to switch roles every 2 minutes during chest compressions*,* coordinating their actions*,* or ensuring proper drug administration. For instance*,* after 5 minutes of cardiac and respiratory arrest*,* the nurse asked the resident*,* ‘Should I administer medication to the patient?’ The resident replied*,* ‘Give one or two doses of epinephrine.’ The nurse then drew 2 to 3 ampoules into a single syringe and administered the medication to the patient at will*,* without considering the appropriate timing for drug administration.”*

Participant No. 13 (Clinical Supervisor/Head Nurse, Cardiac Department) highlighted how some residents’ lack of experience and knowledge led to interference from other team members in leadership roles. She explained:

*“For example*,* regarding team members’ lack of competency: I remember responding to a code in the gynecology ward. The patient developed hypotension and lost consciousness. The team was doing everything except administering norepinephrine. When I insisted*,* they said*,* ‘We don’t even have norepinephrine in this ward!’ I had to urge them to get it from our unit downstairs. The patient stabilized immediately after administration. The ward lacked basic emergency medications*,* and the resident in charge was clearly incompetent—with no clinical knowledge*,* no experience*,* and completely disengaged from the resuscitation. Staff were making arbitrary decisions while the so-called leader ignored everything. “*.

### Participatory leadership

“Participatory Leadership” is one of the dimensions of the concept of team leadership style. Participants noted that skilled, flexible leaders in resuscitation teams consult with team members and accept their logical suggestions. Participant No. 11 said in this regard:

*“Sometimes the team leader says the patient does not indicate atropine*,* but we argue ‘Doctor*,* the patient has a history of bradyarrhythmia*,* and we should administer atropine.’ If the leader has a flexible personality*,* I feel I have a role in this process. However*,* if the leader insists*,* ‘No*,* just administer amiodarone or epinephrine*,*’ then I no longer feel I have a role in the decision-making process.”*

## Discussion

The study aimed to identify the factors influencing interactions among members of cardiopulmonary resuscitation (CPR) teams. Data analysis revealed a main category titled “The Complexity of Cardiopulmonary Resuscitation Interactions.” This included general categories such as: consensus in resuscitation, communication clarity, interaction in team rotation, personal conflicts, and team leadership style. These categories reflected team members’ experiences and the factors influencing their interactions during CPR.

“Consensus in Resuscitation” plays a key role in team interactions, serving as a facilitator of effective teamwork during cardiopulmonary resuscitation (CPR). After participating in CPR cases, team members should debrief and exchange ideas about how to perform their roles in the form of training classes or regular meetings. Studies by Marcino and Gordon highlight the importance of “hot wash” or immediate debriefing sessions—activities conducted right after emergency incidents to document initial impressions, identify challenges, and develop solutions for current and future issues [30]. Research has demonstrated that such debriefings not only enhance the quality of cardiac arrest care in emergency departments but also contribute to improved clinical outcomes, educational growth, and psychological well-being among staff [31–33].

Pre-resuscitation coordination refers to meetings held before cardiopulmonary resuscitation (CPR) to review roles, responsibilities, and necessary processes. These meetings not only improve team preparedness but also significantly increase the chances of successful resuscitation outcomes. As highlighted in the study by Nallamothu et al., hospitals often assign designated resuscitation teams for each shift. However, team members typically have other clinical duties during their shifts and must temporarily abandon those tasks when responding to a resuscitation event. Furthermore, the composition of the team may vary between CPR cases, making it difficult for members to recognize and resolve potential issues in team dynamics. To overcome this challenge, the study emphasizes the importance of holding pre-resuscitation meetings to ensure that all team members are aligned and prepared for their roles [34].

In the participants’ experiences, communication clarity during resuscitation was a significant factor influencing teamwork interactions. Regular communication serves as a facilitating factor for team interactions, while irregular communication serves as a barrier. When resuscitation team members maintain regular, structured communication, they can perform tasks correctly and on time. Clear information exchange ensures all members understand the resuscitation method and process, leading to better team interaction and coordination. Conversely, irregular communication among team members may create incoherence in teamwork, potentially endangering both personnel and patient safety. Indeed, irregular communication is recognized as one of the key impediments to effective team interactions. A study by Bogenstätter et al. examined how resuscitation team members shared information with medical professionals during a simulation. The study found that 18% of the information shared was incorrect, with quantitative information (such as defibrillation counts and drug doses) being more prone to errors. This type of data is particularly vulnerable to mistakes because it can change over time and requires precise communication [35]. Supporting this, Hunziker et al. emphasized the importance of clear communication in supporting teamwork during resuscitation. For example, using specific and clear phrases like, “This is the first defibrillation at 120 J,” helps team members recall critical details and stay aligned during the resuscitation process [36].

The concept of “Interaction in Team Rotation” is another critical factor influencing cardiopulmonary resuscitation (CPR) team interactions. Participants emphasized the importance of correct and timely role assignment and information exchange during role rotation in CPR to prevent ineffective performance and disruptions in teamwork. Team interaction during role rotation can occur as either a normal/facilitating process—where members switch roles promptly and share critical patient information, ensuring effective chest compressions while preventing staff fatigue—or, conversely, as an abnormal one that hinders interactions. In such dysfunctional rotations, members may switch roles without sharing key information or following time standards, often due to power dynamics or responsibility reluctance. This can lead to compromised care, including poor compressions and medication errors. The American Heart Association (AHA) recommends that rescuers performing chest compressions switch roles every two minutes during cardiopulmonary resuscitation (CPR). This practice helps reduce fatigue and ensures that chest compressions remain effective and high-quality [2]. According to the study by Reyes et al. Fatigue from chest compressions over 2 min depends on several factors. These include the rescuers’ traits, education, work experience, and the conditions during CPR [37]. Additionally, Giri et al. emphasized in their study the importance of clear role definition within CPR teams. They stressed that individuals must be aware of their specific roles to perform their duties promptly and effectively [38].

The concept of “Personal Conflicts” is one of the factors that hinder effective interaction within the cardiopulmonary resuscitation (CPR) team. This study identified personal conflicts and a lack of CPR skills as major barriers to effective team interaction. Interpersonal conflicts may occur either before or during CPR team resuscitation efforts. Studies have shown that if conflicts between healthcare professionals are not addressed, conflicts have negative effects on healthcare teams and the quality and safety of patient care, as they can distract providers from patient care [38, 39]. Conflicts within teams can include harsh language (threats, shouting, swearing), blaming, impaired communication, or disruptive behavior [40, 41]. According to the study by Kim et al., conflicts arise when the goals, expectations, and interests of individuals are perceived as incompatible. Conflicts arise between healthcare professionals in multiple specialties and roles, as well as within teams at different stages of patient care. Unresolved conflicts between healthcare professionals can lead to difficult patient care outcomes [42].

“Team Leadership Style” is an important factor in CPR team interactions. Leadership style refers to how a leader motivates, guides, and influences team members to achieve shared goals [43]. The leadership style depends on many factors. These include the manager’s personality, values, skills, and experiences. Internal and external organizational factors also play a role [44]. These elements collectively influence how effective a leader can be in guiding their team and achieving desired outcomes. This study identified autocratic leadership as a significant limiting factor in team interaction quality. Participants reported that the leader of the cardiopulmonary resuscitation (CPR) team rarely consulted them or sought their input. This lack of inclusion suppressed other team members from participating in decision-making, ultimately hindering effective teamwork and collaboration. Thomas’s study supports this, showing that poor leadership and communication in CPR teams contribute to 70% of perinatal deaths and injuries [45]. While these findings highlight the drawbacks of autocratic leadership in team dynamics, empirical evidence from medical teams demonstrates that in critical, time-sensitive contexts (e.g., emergency resuscitation), autocratic leadership enhances performance by accelerating decision-making and reducing errors [46]. This suggests a situational duality: although autocratic approaches may optimize immediate task execution, they can simultaneously undermine team engagement and long-term collaboration. The study by Giltinane highlights that autocratic leaders tend to focus on maintaining power. They are often close-minded and controlling. Because of this, some people may find it hard to work with them. Some nursing staff dislike these leaders for demanding obedience, loyalty, and strict rule-following. However, others thrive under autocratic leadership [47]. This underscores the critical importance of context and individual preferences in determining leadership effectiveness. The laissez-faire leadership style negatively affects team interactions, as demonstrated in this study. Leaders who adopt this style often exhibit indifference and fail to provide adequate supervision or guidance to their team members. This lack of involvement can lead to significant challenges in coordination and cohesion within the team. Inexperienced leaders often use this style. It is also common among those ready to leave their roles. They prefer to let others take charge [48]. The study by Easterby-Smith et al. highlights that laissez-faire leadership is often viewed unfavorably, with many not even considering it a true leadership style due to its lack of direction and engagement [49]. In contrast, the participatory leadership style emerged as a key factor enhancing team interactions in participants’ experiences. In this style, the team leader welcomes logical ideas and input from team members, fostering a collaborative environment. This approach not only enhances teamwork but also increases the likelihood of successful resuscitation efforts. The World Health Organization (WHO) study further supports this, emphasizing that participatory leadership values knowledge, experience, skills, and innovation in decision-making. This leadership style relies on the diverse expertise of team members and their active engagement to achieve optimal results [48].

### Limitations

Like most qualitative research, this study’s findings are context-specific and may not be generalizable to other settings. We recommend replicating this study in private hospital settings, given potential differences in working conditions, organizational protocols, and institutional structures that may influence team dynamics.

### Ethical considerations

This study is part of a doctoral thesis in nursing. It has an ethics code, IR.GUMS.REC.1402.445, from the Ethics Committee at Guilan University of Medical Sciences. The consent form stated that participation was voluntary. Participants got information both verbally and in writing about the study. They could also leave at any time. The researchers gave participants numbers like 1, 2, and 3. They stored the pseudonymization list separately from transcribed data.

## Conclusion

The findings of this study demonstrate that the complex dynamics of resuscitation team interactions are influenced by various barriers and facilitators. To optimize teamwork, we must strategically enhance facilitating factors—including consensus in resuscitation, regular communication, normal rotation, and participatory leadership—while mitigating barriers such as irregular communication, personal conflicts, abnormal rotation, and autocratic leadership. This means that if members of the CPR team collaborate effectively, avoid personal conflicts, and reach consensus on their roles and tasks during resuscitation while adopting a participatory leadership style, they can foster better interactions. Additionally, maintaining clear communication, ensuring timely role transitions, and avoiding individualism during chest compressions further enhances teamwork. By addressing these factors, we can achieve optimal team interaction, ultimately leading to successful cardiopulmonary resuscitation.

## Supplementary Information

Supplementary Material 1.

## Data Availability

Data are available by contacting the corresponding author.

## Abbreviations

CPR: Cardiopulmonary Resuscitation
IHCA: In-hospital cardiac arrest
COREQ: Consolidating Criteria for Reporting Qualitative Research
ICU: Intensive care unit
CCU: Cardiac care unit
AHA: American Heart Association
WHO: World Health Organization
GUMS: Guilan University of Medical Sciences

## References

[1] Giacoppo DJEHJ. Impact of bystander-initiated cardiopulmonary resuscitation for out-of-hospital cardiac arrest: where would you be happy to have a cardiac arrest? Eur Heart J. 2019;40(3):319–21. 10.1093/eurheartj/ehy911. 30649371 10.1093/eurheartj/ehy911

[2] Panchal AR, Bartos JA, Cabañas JG, Donnino MW, Drennan IR, Hirsch KG, et al. Part 3: adult basic and advanced life support: 2020 American heart association guidelines for cardiopulmonary resuscitation and emergency cardiovascular care. Circulation. 2020;142(16Suppl2):S366–468. 33081529 10.1161/CIR.0000000000000916

[3] Abdollahi H, Tayebeh P, Mazlum R, Malekzadeh J, Farsi M, Janati FJ. Effect of capnography feedback during CPR on return of spontaneous circulation. Med J Islam Repub Iran. 2018;61(1):816–24. 10.22038/mjms.2018.11194.

[4] Clarke S, Apesoa-Varano EC, Barton J. Code blue: methodology for a qualitative study of teamwork during simulated cardiac arrest. BMJ Open. 2016;6(1):e009259. 26758258 10.1136/bmjopen-2015-009259 PMC4716199

[5] Finke S-R, Schroeder DC, Ecker H, Wingen S, Hinkelbein J, Wetsch WA, et al. Gender aspects in cardiopulmonary resuscitation by schoolchildren: A systematic review. Resuscitation. 2018;125:70–8. 10.1016/j.resuscitation.2018.01.025. 29408490 10.1016/j.resuscitation.2018.01.025

[6] Goodarzi A, Jalali A, Almasi A, Naderipour A, Kalhori RP, Khodadadi AJG. Study of survival rate after cardiopulmonary resuscitation (CPR) in hospitals of Kermanshah in 2013. Glob J Health Sci. 2014;7(1):52. 25560341 10.5539/gjhs.v7n1p52 PMC4796346

[7] Chopra A, Wong N, Ziegler C, Morrison LJR. Systematic review and meta-analysis of hemodynamic-directed feedback during cardiopulmonary resuscitation in cardiac arrest. Resuscitation. 2016;101:102–7. 26875990 10.1016/j.resuscitation.2016.01.025

[8] Neumar RW, Shuster M, Callaway CW, Gent LM, Atkins DL, Bhanji F, et al. Part 1: executive summary: 2015 American heart association guidelines update for cardiopulmonary resuscitation and emergency cardiovascular care. Circulation. 2015;132(18suppl2):S315–67. 26472989 10.1161/CIR.0000000000000252

[9] Silverplats J, Strömsöe A, Äng B. Södersved Källestedt M-LJPo. Attitudes towards cardiopulmonary resuscitation situations and associations with potential influencing factors—A survey among in-hospital healthcare professionals. PLoS ONE. 2022;17(7):e0271686. 35839233 10.1371/journal.pone.0271686 PMC9286263

[10] Ghasemi A, Rezapour-Nasrabad R, Mokhlesabadifarahan T, Alizadeh Z, Beygi N, Fereidouni Z, et al. Exploring the challenges affecting the quality of cardiopulmonary resuscitation from the perspective of emergency medical service personnel: a qualitative study. BMC Emerg Med. 2021;40(2):154–63.

[11] Matsui S, Kurosawa H, Hayashi T, Takei H, Tanizawa N, Ohnishi Y, Murata S, Ohnishi M, Yoshii TH, Miyawaki K, Matsumoto T. Annual patterns in the outcomes and post-arrest care for pediatric out-of-hospital cardiac arrest: A nationwide multicenter prospective registry in Japan. Resuscitation. 2023;191:109942. 37625577 10.1016/j.resuscitation.2023.109942

[12] Salas E, Diaz-Granados D, Klein C, Burke CS, Stagl KC, Goodwin GF, et al. Does team training improve team performance? A meta-analysis. Hum Factors. 2008;50(6):903–33. 19292013 10.1518/001872008X375009

[13] Porter JE, Cant RP, Cooper SJ. Rating teams’ non-technical skills in the emergency department: A qualitative study of nurses’ experience. Int Emerg Nurs. 2018;38:15–20. 29422222 10.1016/j.ienj.2017.12.006

[14] Lauridsen KG, Krogh K, Müller SD, Schmidt AS, Nadkarni VM, Berg RA, Bach L, Dodt KK, Maack TC, Møller DS, Qvortrup M. Barriers and facilitators for in-hospital resuscitation: a prospective clinical study. Resuscitation. 2021;164:70–8. 34033863 10.1016/j.resuscitation.2021.05.007

[15] Krajina I, Kvolik S, Steiner R, Kovacevic K, Lovric I. Cardiopulmonary resuscitation, chest compression only and teamwork from the perspective of medical doctors, surgeons and anesthesiologists. Iran Red Crescent Med J. 2015;17(3):e18208. 26019895 10.5812/ircmj.18208 PMC4441776

[16] Pfaff J, Moore G. Rosen’s Emergency Medicine: Concepts and Clinical Practice. Otolaryngology chapter. Mastoiditis. Philadelphia: Elsevier Publishing; 2018. https://www.elsevier.com/books/rosens-emergency-medicine/marx/978-0-323-77984-2.

[17] Saunders R, Wood E, Coleman A, Gullick K, Graham R, Seaman K. Emergencies within hospital wards: an observational study of the non-technical skills of medical emergency teams. Australasian Emerg Care. 2021;24(2):89–95. 10.1016/j.auec.2020.07.003 32747297

[18] Hosseini M, Heydari A, Reihani H, Kareshki H. Elements of teamwork in resuscitation: an integrative review. Bull Emerg Trauma. 2022;10(3):95. 35991377 10.30476/BEAT.2021.91963.1291 PMC9373058

[19] Calder LA, Mastoras G, Rahimpour M, Sohmer B, Weitzman B, Cwinn AA, et al. Team communication patterns in emergency resuscitation: a mixed methods qualitative analysis. Int J Emerg Med. 2017;10:1–9. 28707273 10.1186/s12245-017-0149-4 PMC5509566

[20] Gabr AK. The importance of nontechnical skills in leading cardiopulmonary resuscitation teams. J Royal Coll Physicians Edinb. 2019;49(2):112–6. 10.4997/JRCPE.2019.205 31188338

[21] Chalwin R, Flabouris AJI. Utility and assessment of non-technical skills for rapid response systems and medical emergency teams. Intern Med J. 2013;43(9):962–9. 23611153 10.1111/imj.12172

[22] Briggs A, Raja AS, Joyce MF, Yule SJ, Jiang W, Lipsitz SR, et al. The role of nontechnical skills in simulated trauma resuscitation. J Surg Res. 2015;72(4):732–9. 10.1016/j.jsurg.2015.01.020 25817012

[23] Rosenman ED-, Rosenman ED, Bullard MJ, Jones KA, Welsh L, Brolliar SM, Levine BR, Grand JA, Fernandez R. Development and empirical testing of a novel team leadership assessment measure: a pilot study using simulated and live patient encounters. AEM Educ Train. 2019;3(2):163–71. 31008428 10.1002/aet2.10321 PMC6457354

[24] Chan PS, Nallamothu BKJJ. Improving outcomes following in-hospital cardiac arrest: life after death. JAMA. 2012;307(18):1917–8. 22570458 10.1001/jama.2012.3504 PMC3517892

[25] Weaver SJ, Dy SM. Rosen majbq, safety. Team-training in healthcare: a narrative synthesis of the literature. BMJ Qual Saf. 2014;23(5):359–72. 24501181 10.1136/bmjqs-2013-001848 PMC3995248

[26] Davoodvand S, Abbaszadeh A, Ahmadi F. Patient advocacy from the clinical nurses’ viewpoint: a qualitative study. J Med Ethics History Med. 2016;9:5. PMC4958925 27471588

[27] Elo S, Kyngäs H. The qualitative content analysis process. J Adv Nurs. 2008;62(1):107–15. 18352969 10.1111/j.1365-2648.2007.04569.x

[28] Polit D, Beck C. Essentials of nursing research: Appraising evidence for nursing practice. Philadelphia: Lippincott Williams & Wilkins; 2020.

[29] Dworkin SL. Sample size policy for qualitative studies using In-Depth interviews. Arch Sex Behav. 2012;41(6):1319–20. 22968493 10.1007/s10508-012-0016-6

[30] Marcino D, Gordon KJCCDR. Emergency response: an overview of the National microbiology laboratory emergency management program. Can Commun Dis Rep. 2018;44(5):102. 31007619 10.14745/ccdr.v44i05a02 PMC6449121

[31] Nocera M. Merritt cjae, training. Pediatric critical event debriefing in emergency medicine training: an opportunity for educational improvement. AEM Educ Train. 2017;1(3):208–14. 30051036 10.1002/aet2.10031 PMC6001495

[32] Schmidt M, Haglund KJJoTN JTN. Debrief in emergency departments to improve compassion fatigue and promote resiliency. J Trauma Nurs. 2017;24(5):317–22. 28885522 10.1097/JTN.0000000000000315

[33] Gilmartin S, Martin L, Kenny S, Callanan I, Salter NJB. Promoting hot debriefing in an emergency department. BMJ Open Qual. 2020;9(3):e000913. 32816864 10.1136/bmjoq-2020-000913 PMC7430325

[34] Nallamothu BK, Guetterman TC, Harrod M, Kellenberg JE, Lehrich JL, Kronick SL, et al. How do resuscitation teams at top-performing hospitals for in-hospital cardiac arrest succeed? A qualitative study. Circulation. 2018;138(2):154–63. 29986959 10.1161/CIRCULATIONAHA.118.033674 PMC6245659

[35] Bogenstätter Y, Tschan F, Semmer NK, Spychiger M, Breuer M, Marsch SJH. How accurate is information transmitted to medical professionals joining a medical emergency? A simulator study. Hum Factors. 2009;51(2):115–25. 19653477 10.1177/0018720809336734

[36] Hunziker S, Johansson AC, Tschan F, Semmer NK, Rock L, Howell MD, et al. Teamwork and leadership in cardiopulmonary resuscitation. J Am Coll Cardiol. 2011;57(24):2381–8. 21658557 10.1016/j.jacc.2011.03.017

[37] Reyes M, Díaz C, Cuineme SJJCCR. Quality CPR rescuer alternating every 2 minutes 1 minute vs university hospital clinic in San rafael, 2018. J Crit Care Res. 2019;12(4):89–94.

[38] Giri S, Gyeltshen D, Wangchuk N, Dorji K, Drakpa L, Wangdi S, et al. Improving role allocation for cardiopulmonary resuscitation (CPR) in the emergency department: a quality improvement project. BMJ Open Qual. 2024;13(3):e002870. 10.1136/bmjoq-2024-002870. 10.1136/bmjoq-2024-002870 PMC11448221 39349305

[39] De Wit FR, Greer LL, Jehn KAJJoap. The paradox of intragroup conflict: a meta-analysis. J Appl Psychol. 2012;97(2):360. 21842974 10.1037/a0024844

[40] Azoulay E, Timsit J-F, Sprung CL, Soares M, Rusinova K, Lafabrie A, et al. Prevalence and factors of intensive care unit conflicts: the conflicts study. Am J Respir Crit Care Med. 2009;180(9):853–60. 19644049 10.1164/rccm.200810-1614OC

[41] Rogers D, Lingard L, Boehler ML, Espin S, Klingensmith M, Mellinger JD, et al. Teaching operating room conflict management to surgeons: clarifying the optimal approach. Med Educ. 2011;45(9):939–45. 21848722 10.1111/j.1365-2923.2011.04040.x

[42] Kim S, Bochatay N, Relyea-Chew A, Buttrick E, Amdahl C, Kim L, et al. Individual, interpersonal, and organisational factors of healthcare conflict: A scoping review. J Interprof Care. 2017;31(3):282–90. 28276847 10.1080/13561820.2016.1272558

[43] Mosadeghrad AM. Ferdosi MJMs-m. Leadership, job satisfaction and organizational commitment in healthcare sector: proposing and testing a model. Mater Sociomed. 2013;25(2):121. 24082837 10.5455/msm.2013.25.121-126 PMC3769150

[44] Mosadeghrad AJTDT. Essentials of healthcare organization and management. Tehran: Dibagran Tehran; 2015.

[45] Thomas E, Taggart B, Crandell S, Lasky R, Williams A, Love L, et al. Teaching teamwork during the neonatal resuscitation program: a randomized trial. J Perinatol. 2007;27(7):409–14. 17538634 10.1038/sj.jp.7211771

[46] Künzle B, Kolbe M, Grote G. Ensuring patient safety through effective leadership behaviour: a literature review. Saf Sci. 2010;48(1):1–7.

[47] Giltinane CLJNs. Leadership styles and theories. Nurs Stand. 2013;27(41):35–9. 10.7748/ns2013.06.27.41.35.e7565 23905259

[48] World Health Organization. Open mindsets: participatory leadership for health. InOpen mindsets: participatory leadership for health 2016. https://iris.who.int/handle/10665/251458.

[49] Easterby-Smith M, Thorpe R, Jackson PR. Management research, vol. 3. London: Sage; 2012.

