# Supplementary material for: Barriers and facilitators for interaction in cardiopulmonary resuscitation teams: a qualitative study
Source: Int J Emerg Med. 2025 Jul 28;18:134. doi: 10.1186/s12245-025-00955-8 (PMC12302452; doi:10.1186/s12245-025-00955-8)
Supplement: Supplementary file 1 — Supplementary Material 1. [file 12245_2025_955_MOESM1_ESM.docx]

**Interview Guide: Teamwork Dynamics in CPR**

Purpose: To understand team interactions, facilitators, and barriers during resuscitation efforts.

1. General Open-Ended Questions

"What has been your experience with teamwork during cardiopulmonary resuscitation?"

2. Team Roles & Interactions

" How would you describe interactions within your CPR team?"

"How are roles typically assigned during CPR?

"Can you share an example of effective role clarity (or confusion)?"

"How do you experience the communication between the team leader and members during resuscitation?

Could you describe a specific example of this dynamic?"

"What communication strategies have you observed that enhance team coordination during resuscitation?"

3. Facilitators & Barriers of Effective Teamwork

" What factors have you observed affecting CPR team interactions?"

"What challenges have you faced in CPR teamwork?

"Can you recall a time when interpersonal conflicts affected resuscitation? How was it addressed?"

What debriefing practices help your team reflect on performance?"

Probing prompts: Encourages specific examples (e.g., "Can you share an instance when...").
